# miRNA-150_R-1 mediates the HIF-1/ErbB signaling pathway to regulate the adhesion of endometrial epithelial cells in cows experiencing retained placenta

**DOI:** 10.3389/fvets.2022.1037880

**Published:** 2022-10-17

**Authors:** Chen Lv, Zongshuai Li, Qi Wang, Yue Wang, Xingxu Zhao, Yong Zhang

**Affiliations:** ^1^College of Veterinary Medicine, Gansu Agricultural University, Lanzhou, China; ^2^Gansu Key Laboratory of Animal Generational Physiology and Reproductive Regulation, Lanzhou, China

**Keywords:** cattle, retained placenta, miR-150_R-1, EPAS1, HIF-1/ErbB signaling pathway

## Abstract

Retained placenta (RP) refers to reproductive disorders caused by the failure of fetal membranes to be expelled 12 h after delivery in dairy cows. Postpartum adhesion of the fetal membranes to the uterus causes diseases such as mastitis or endometritis, which threatening the profitability of the dairy industry. Emerging evidence suggests that micro RNAs (miRNAs) play crucial roles in various processes, such as the occurrence and progression of fetal membranes discharge. However, the molecular mechanisms of miRNAs in RP remain unknown. In this study, we performed RNA-sequencing to characterize the expression profiles of mRNAs and miRNAs in caudal vein blood samples of postpartum Holstein cows whose fetal membranes were discharged normally or retained to identify RP-related genes and evaluate their molecular mechanisms. We identified 44 differentially expressed miRNAs (19 upregulated and 25 downregulated) and 706 differentially expressed mRNAs (325 upregulated and 381 downregulated) in the RP group compared to the normal fetal membranes discharge group. Gene Ontology and Kyoto Encyclopedia of Genes and Genomes analysis revealed that differentially expressed mRNAs were mainly enriched in the extracellular matrix, cell adhesion, and autoimmunity-related biological processes or pathways. Further analyses using RNA-sequencing, a dual luciferase reporter system, quantitative reverse transcription-PCR, immunofluorescence, and western blotting verified that endothelial PAS domain protein 1 (EPAS1) is regulated by miR-150_R-1 in endometrial epithelial cells. We demonstrated the relationship between EPAS1 and RP and confirmed that EPAS1 is upregulated in the blood and placenta of cows that experience RP. Further, we proposed a model of the miRNA-mRNA negative regulatory network mediated by the HIF-1/ErbB signaling pathway to show its regulatory role in RP.

## Introduction

Retained placenta (RP) is a pathological adhesion between the fetal cotyledons and maternal uterine caruncle (1). During pregnancy, endometrial epithelial and trophoblast ectoderm cells continuously migrate and fuse, and fetal villi are embedded in the maternal uterine caruncle to form a placenta with a tightly connected epithelial connective chorionic structure (2). Normally, after delivery, the microvilli of the fetal cotyledon easily separate from the caruncle of the maternal uterus (3). However, it is difficult to separate the mutual adhesion between the fetal cotyledon and uterine caruncle, which typically involves the adhesion of key cells constituting the placental tissue, leading to RP. When some or all fetal membranes adhere to the uterus, they eventually degrade and cause diseases such as mastitis or endometritis, resulting in large economic losses in the dairy industry (4).

miRNAs are endogenous non-coding RNAs ~18–25 nucleotides (nt) in length and exert regulatory functions (5). miRNAs bind to the 3′-untranslated (UTR) region complementary to the mRNA sequence and correspondingly inhibit or repress mRNA translation (6). Recent studies showed that various miRNAs play major roles in the molecular mechanism of placental development (5–7). In addition, miRNAs are involved in the biological functions of trophoblast and endometrial epithelial cells (8–10). For example, Zheng et al. found that miRNA-185 regulates endometrial epithelial cells by targeting stromal interaction molecule 1 (STIM1) (9) and that miRNA-185 mediates the vascular endothelial growth factor (VEGF) A signaling pathway to regulate RP (10). However, studies are needed to determine the regulatory mechanism of miRNAs in fetal membranes shedding to positively affect the dairy industry.

RNA-sequencing (RNA-seq) is used to identify mRNAs and miRNAs expressed in mammalian tissues and blood (11). This method can also be performed for in-depth screening of mRNA-miRNAs associated with RP and to understand the complex pathogenesis of this disease. In this study, RNA-seq analysis and molecular biology experiments were conducted to screen and identify differentially expressed (DE) genes in the blood of the fetal membranes that are normally discharged or retained. We also investigated whether the genes involved in RP are targeted and regulated by miRNAs and whether the alteration of the expression of related molecules in the signaling pathway by these genes can predict the pathogenesis of RP.

## Materials and methods

### Experimental samples

The Animal Protection Committee of Gansu Agricultural University (Lanzhou, China) approved all animal experiments (Approval No.: GSAU-Eth-LST-2021-003). Holstein cows of similar age, parity, and weight (age 3–4 years; 2–3 parities; 500 ± 10 kg) were selected from a feeding environment without other diseases at an intensive breeding farm in Zhangye City, Gansu Province. The cows were divided into two groups: normal discharge of fetal membranes (NC; *n* = 3) and the failure of fetal membranes to be expelled 12 h after delivery (RP; *n* = 3). Blood samples collected from the caudal vein and placenta tissues of the NC and RP groups were labeled as NC-1, NC-2, and NC-3 and RP-1, RP-2, and RP-3, respectively. The collected tissues were washed with sterile saline. The tissues and whole blood samples were snap-frozen in liquid nitrogen for 30 min and stored at −80°C. The remaining placental tissue was fixed in 4% paraformaldehyde (Biosharp, Anhui, Hefei, China) or 2.5% glutaraldehyde solution (Servicebio, Hubei, Wuhan, China) and stored at 4°C.

### Hematoxylin-eosin staining

Paraffin sections of the placental tissue were prepared using a routine method of dewaxing with xylene, dehydration through an alcohol gradient, staining with hematoxylin and eosin, dehydration through an alcohol gradient, dewaxing with xylene, and sealing with neutral balsam. Three visual fields were randomly selected and viewed under a microscope (Olympus, Tokyo, Japan) for histopathological analyses.

### Transmission electron microscopy

Placental samples were presoaked in a 2.5% glutaraldehyde solution. The samples were soaked in 1% osmium tetroxide, dehydrated through an alcohol gradient, immersed in propylene oxide transition fluid, and double-stained with uranyl acetate-lead citrate. A randomly selected field of view was photographed under a Hitachi HT7700 transmission electron microscope (Hitachi, Tokyo, Japan); this process was repeated three times.

### Identification of miRNAs and mRNAs in cattle blood by RNA-seq

Total RNA from the NC and RP groups was isolated and purified using TRIzol reagent (Thermo Fisher Scientific, Waltham, MA, USA). The total RNA was used as input material to synthesize miRNA and mRNA libraries using the TruSeq Small RNA Sample Prep Kit and Ribo-Zero™ rRNA Removal Kit (Illumina, San Diego, CA, USA) according to the manufacturer's instructions. Fragments per kilobase of exon per million mapped fragments were calculated to determine the miRNA and mRNA expression levels using StringTie (12). The miRNAs and mRNAs with |log2 fold-change (FC)| > 1 and that were statistically significant (*p* < 0.05) were selected as DE genes (13). The functions of the DE mRNAs and miRNAs were analyzed using Gene Ontology (GO) and Kyoto Encyclopedia of Genes and Genomes (KEGG) with the DAVID online tool (https://david.ncifcrf.gov/summary.jsp).

### Cell culture and transfection

The bovine endometrial epithelial cell line (BEND) was donated by Prof. Dong Weitao of the College of Veterinary Medicine, Gansu Agricultural University. The cell line was checked for mycoplasma contamination using PCR. BEND cells were cultured in DEME/F-12 medium supplemented with 10% fetal bovine serum (Invitrogen, Carlsbad, CA, USA) and 1% penicillin/streptomycin (Solarbio, Beijing, China) in humidified air at 37°C in a 5% CO_2_ incubator (Thermo Fisher Scientific). BEND cells were seeded (4 × 10^5^ cells/well) into 6-well plates in complete medium and allowed to settle overnight. When the cells were 50–80% confluent, they were transfected with miR-150_R-1 mimic or mimic NC at a final concentration of 25, 50, or 100 nM using proportional doses of Lipofectamine 2000 (Invitrogen). Pre-miR-transfected cells were harvested at different time points (12, 24, 48, or 72 h) after transfection.

### Quantitative reverse transcription-PCR

RNase-free consumables were used during RNA extraction. TRIzol reagent was used to extract the total RNA from blood, placental tissue, and cells. First-strand cDNA for mRNA or miRNA was synthesized from 500 ng of each total RNA sample using an Evo M-MLV RT Kit with gDNA Clean for quantitative PCR (qPCR) or miRNA 1st strand cDNA synthesis kit (Accurate Biology, Changsha, Hunan, China) following the manufacturer's instructions, respectively. Quantitative reverse transcription (qRT)-PCR was conducted using TransStart^®^ Green qPCR SuperMix (TransGen Biotech, Beijing, China) on a LightCycler 96 Real-Time Systems (Roche, Basel, Switzerland). Specific primers were designed and synthesized by AZENTA Life Sciences (Tianjin, China) and Sangon Biotech (Shanghai, China). The primer sequences are shown in Supplementary Table S1. β-*Actin* or U6 mRNA levels were measured for normalization. These experiments were performed in triplicate, and relative expression was calculated using the 2^−Δ*ΔCt*^ method (14).

### Luciferase reporter assay

The wild-type (WT) EPAS1 3′-UTR fragment containing the putative miR-150_R-1 binding site and its corresponding mutant type (Mut) were designed and synthesized (AZENTA Life Sciences) and cloned into the pmirGLO cloning vector between the NheI and XhoI cloning sites; the products were named EPAS1-3′-UTR WT and EPAS1-3′-UTR MUT. A miR-150_R-1 mimic (5′-3′: UCUCCCAACCCUUGUACCAGUG, CUGGUACAAGGGUUGGGAGAUU) and mimic NC (5′-3′: UUGUACUACACAAAAGUACUG, GUACUUUUGUGUAGUACAAUU) were purchased from Sangon Biotech. BEND cells were seeded into 24-well plates and co-transfected with luciferase reporter constructs encoded with EPAS1-3′-UTR WT or EPAS1-3′-UTR MUT and miR-150_R-1 mimics or mimics NC using Lipofectamine 2000, according to the manufacturer's instructions. At 12 h after transfection, luciferase activity was detected using a Dual-Luciferase Reporter Assay Kit (Promega, Madison, WI, USA).

### Immunofluorescence

At 12 h after transfection, the cells were fixed in 4% paraformaldehyde for 30 min. After 10 min of permeabilization with 0.5% Triton X-100, the cells were washed with phosphate-buffered saline and blocked with 5% bovine serum albumin for 30 min. The cells were incubated overnight at 4°C with mouse anti-EPAS1 (No. 66731-1-Ig, 1:350, Proteintech, Rosemont, IL, USA). The corresponding secondary antibody fluorescein isothiocyanate-conjugated AffiniPure goat anti-mouse IgG (H+L) (No. SA00003-1, 1:200; Proteintech) was incubated with the cells for 2 h at 37°C. The cell nucleus was stained with DAPI, and the cells were observed under a fluorescence microscope (ECHO, Chicago, IL, USA).

### Western blotting

A BCA kit was used to quantify the total amount of protein extracted from the cells using RIPA buffer containing phenylmethylsulfonyl fluoride (Solarbio). The proteins were separated using sodium dodecyl sulfate polyacrylamide gel electrophoresis and transferred onto polyvinylidene fluoride membranes (Millipore, Billerica, MA, USA). Non-specific binding to the membranes was blocked by incubating the membranes in blocking buffer (Beyotime, Shanghai, China), after which the membranes were incubated with rabbit anti-transforming growth factor-α (TGF-α) (No. GB112570; 1:3,000), rabbit anti-epidermal growth factor receptor (EGFR) (No. GB11084, 1:800), and rabbit anti-SRC (No. GB112343, 1:800) from Servicebio; mouse anti-EPAS1 (1:3,000), mouse anti-focal adhesion kinase (FAK) (No. 66258-1-Ig, 1:4,000), and mouse anti-GAPDH (No. 60004-1-Ig, 1:5,000) from Proteintech; and rabbit anti-phospho-FAK (Y397) (No. ab81298, 1:1,000) from Abcam (Cambridge, UK) overnight at 4°C. Horseradish peroxidase (HRP)-conjugated secondary antibodies were incubated with the membranes for 2 h at 37°C. The secondary antibodies were HRP-conjugated AffiniPure goat anti-mouse IgG (H+L) (No. SA00001-1, 1:10,000), and HRP-conjugated AffiniPure goat anti-rabbit IgG (H+L) (No. SA00001-2, 1:10,000); Proteintech). Finally, a chemiluminescence kit (Beyotime) was used to visualize the membranes. GAPDH expression was detected as an endogenous control. Image-Pro Plus 6.0 (Media Cybernetics, Rockville, MD, USA) was used to quantify and scan the optical densities of the bands. Immunoblotting was performed in triplicate for all samples.

### Statistical analysis

The experimental results are expressed as the mean ± SEM. GraphPad Prism software (version 7.0; GraphPad, Inc., San Diego, CA, USA) was used to analyze the comparisons among groups using Student's *t*-tests and one-way analyses of variance. *p* < 0.05 and *p* < 0.01 were considered to indicate statistically significant and highly statistically significant results, respectively.

## Results

### Placental differences between the NC and RP groups

**Figure 1:**
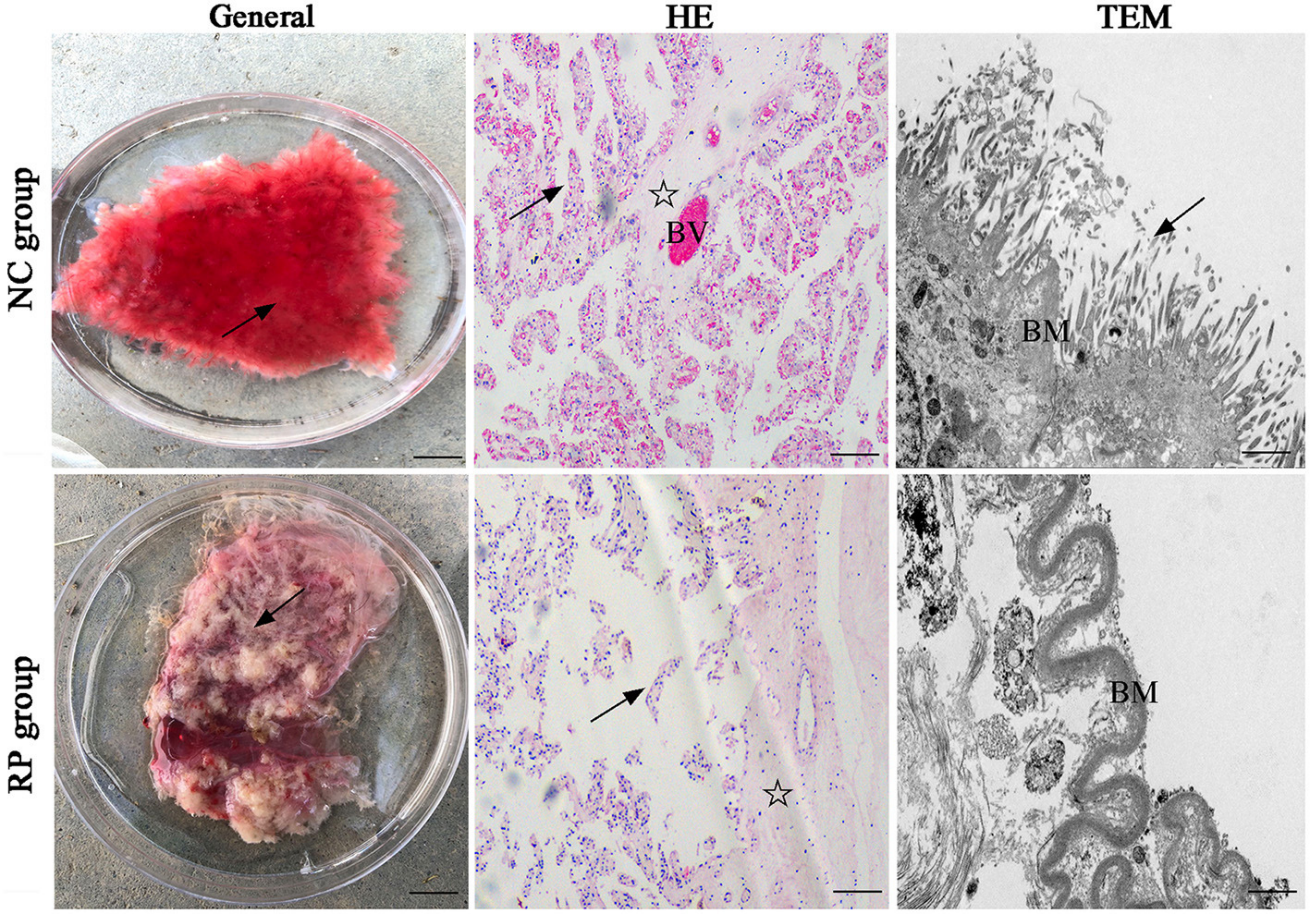
General and pathological differences in the NC and RP groups. General images: scale bar represents 1 cm; HE-stained images: scale bar represents 100 μm; TEM images: scale bar represents 5 μm. Villi (arrow), matrix (asterisk), blood vessel (BV), basement membrane (BM). NC, fetal membranes of normal discharge; RP, retained placenta; HE, hematoxylin-eosin; TEM, transmission electron microscopy.

In the normally discharged fetal membranes, the cotyledons of the placenta were bright red because they were rich in blood vessels (BVs), and the cotyledon villi were arranged in an orderly manner on the surface (Figure 1). The cotyledons of the RP placenta were shrunken because of exposure to air; the color changed from bright red to gray-red, and the villi on the surface were shriveled into clumps.

Hematoxylin-eosin (HE) staining of the sections showed that the normal placentas had highly vascularized cotyledons, with many dilated BVs within the stroma and lamina propria. In contrast, the epithelium detached from the basement membranes (BMs), indicating clear alterations in the RP placentas.

Finally, transmission electron microscopy revealed that the neatly arranged microvilli resembled a brush on the lateral surface of the normally discharged fetal membranes, and the BMs of the villi were intact. The villi in the RP group were short and thin, and the chorionic BMs were thickened and curved.

### Overview of miRNAs sequencing datasets in NC and RP blood

An miRNA library was constructed from the blood of NC and RP cows and sequenced using RNA-seq to investigate the expression of miRNAs in RP cows. Averages of 9,978,400 and 10,025,297 raw reads were obtained from the NC and RP libraries, respectively. After removing adapter dimers, low-complexity, junk reads, common RNA families (ribosomal RNA, tRNA, small nuclear RNA, small nucleolar RNA), and repeats, we obtained averages of 9,161,210 (91.36%) and 9,419,625 (93.96%) high-quality clean reads, representing averages of 134,360 (44.88%) and 80,783 (42.29%) unique sequences of 18–26 nt in the NC and RP libraries, respectively (Supplementary Table S2). Further analysis revealed low levels of large fragments, such as mRNA and ribosomal RNA, indicating high-quality, non-degraded RNA samples. Averages of 91.86 and 95.31% of the small RNAs identified had sizes of 20–24 nt in the NC and RP groups, respectively; these sequences were mainly concentrated at 22 nt (Supplementary Figure S1).

**Figure 2:**
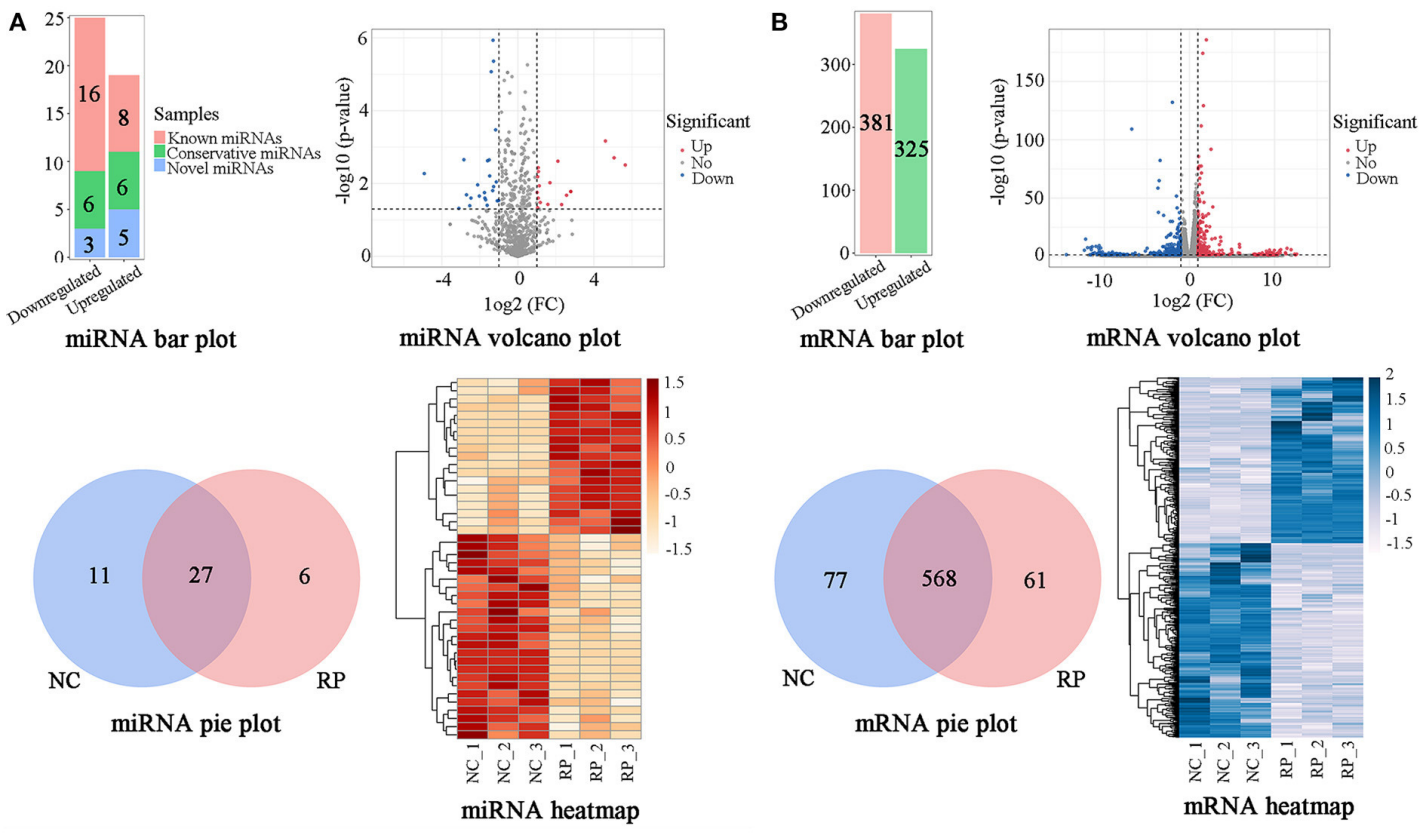
Classification of DE genes in NC and RP. **(A)** Numbers, volcano plot, Venn diagram, and heatmap of DE miRNAs. **(B)** Numbers, volcano plot, Venn diagram, and heatmap of DE mRNAs. The abscissa and ordinate of the heatmap are samples and genes, respectively. The colors from red to white represent the miRNA expression levels [log10 (relative expression levels + 1) and *Z*-score] from high to low. The colors from blue to white represent the mRNA expression levels [log10 (relative expression levels) and *Z*-score] from high to low. DE, differentially expressed; FC, fold-change; NC, fetal membranes of normal discharge; RP, retained placenta.

A BLAST search in miRBase revealed 1,206 miRNAs classified into five categories (Supplementary Table S3). According to the criteria of *p* < 0.05 and |log2 FC| > 1, comparison of the NC and RP groups showed that 44 genes were significantly differentially expressed (Figure 2A; Supplementary Table S4). Of these, 19 were upregulated and 25 downregulated in the NC samples compared to in the RP samples; 24 known miRNAs, 12 conservative miRNAs, and 8 novel miRNAs were identified. There were 27 co-expressed genes between the NC and RP groups, 11 unique genes in NC, and 6 unique genes in RP. A heatmap of the DE miRNAs showed excellent repeatability in the NC and RP groups.

### Overview of mRNAs sequencing datasets in NC and RP blood

An mRNA library was constructed from the blood of NC and RP cows and sequenced using RNA-seq to investigate the expression of mRNAs in RP cows. Averages of 87,901,020 and 87,978,289 raw reads were obtained for the NC and RP libraries, respectively. After removing low-quality sequences, we obtained averages of 78,739,864 (89.58%) and 79,922,722 (90.85%) high-quality clean reads from the NC and RP libraries, respectively (Supplementary Table S5). Of the total clean reads, 91.09% and 91.74% were mapped to the reference genomes for NC and RP, respectively. These results suggest that the clean reads were well-mapped to the genome reference sequences.

Using the criteria of *p* < 0.05 and |log2 FC| > 1, comparison of NC and RP showed that 706 genes were significantly DE (Figure 2B; Supplementary Table S6). Of the 706 DE miRNAs, 325 were upregulated and 381 were downregulated in the NC group compared to the RP group. There were 568 genes co-expressed between the NC and RP groups, 77 unique genes in NC, and 61 unique genes in RP. A heatmap of the DE mRNAs showed excellent repeatability for the NC and RP groups.

### Functional analysis of miRNA-mRNA

**Figure 3:**
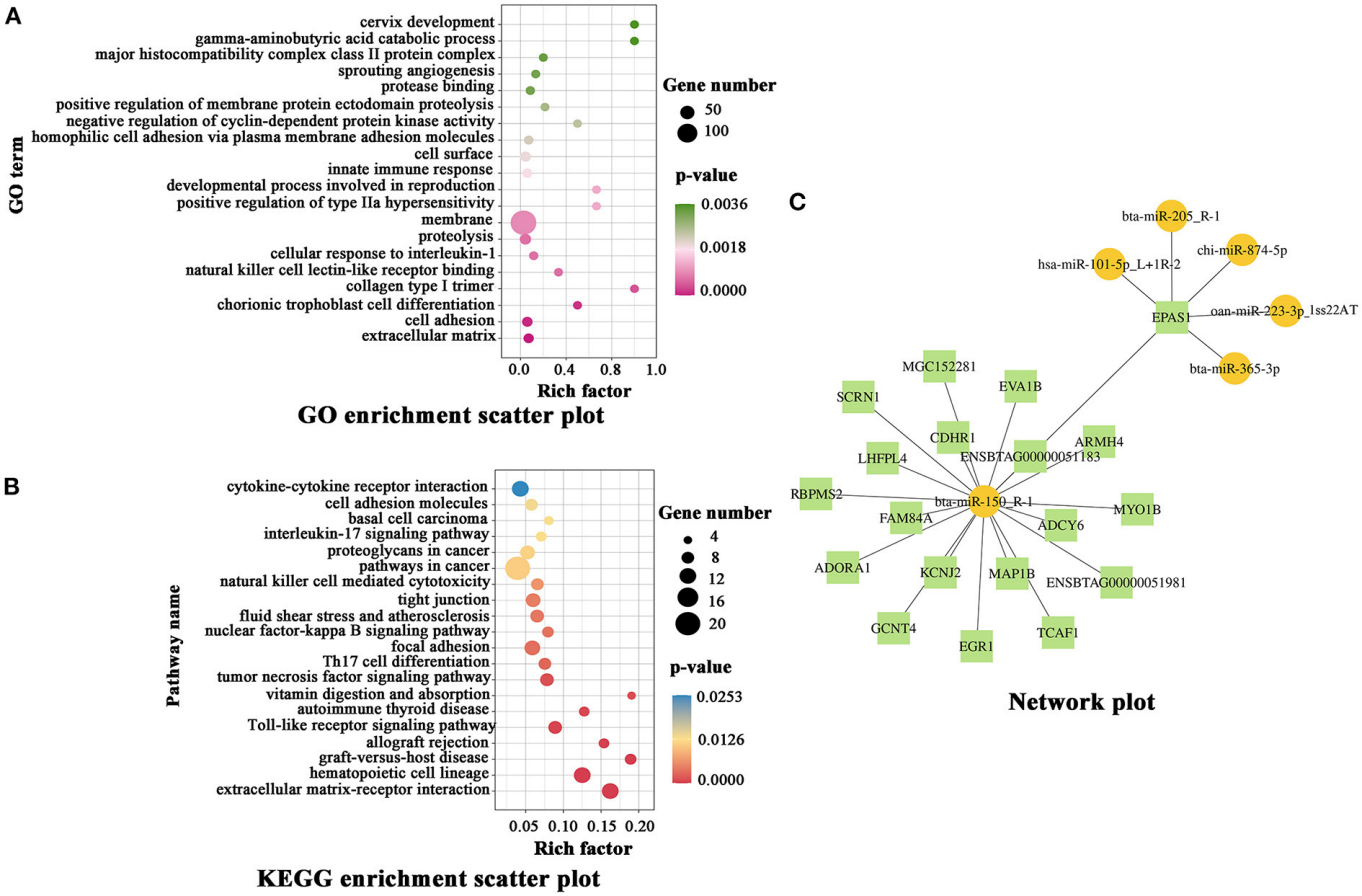
Functional analysis of DE genes in NC and RP. **(A)** GO annotation of DE mRNAs. **(B)** KEGG enrichment analysis of DE mRNAs. **(C)** miR-150_R-1 and target gene *EPAS1* network plot; yellow spheres and green squares represent miRNA and mRNA, respectively; lines represent the interaction between miRNAs/mRNAs. GO, Gene Ontology; KEGG, Kyoto Encyclopedia of Genes and Genomes; NC, fetal membranes of normal discharge; RP, retained placenta.

Functional analysis of the mRNA-miRNAs was performed to screen for RP-associated DE genes. Detailed information on the DE miRNAs and mRNAs is shown in Supplementary Tables S4, S6. GO analysis showed that the DE genes were mainly involved in the extracellular matrix, cell adhesion, chorionic trophoblast cell differentiation, collagen type I trimer, natural killer cell lectin-like receptor binding, cellular response to interleukin-1, proteolysis, membrane, positive regulation of type IIa hypersensitivity, developmental process involved in reproduction, and other biological processes (Figure 3A). KEGG enrichment analysis showed that the DE mRNAs were mainly involved in pathways related to extracellular matrix-receptor interaction, hematopoietic cell lineage, graft-versus-host disease, allograft rejection, Toll-like receptor signaling pathway, autoimmune thyroid disease, vitamin digestion and absorption, tumor necrosis factor signaling pathway, Th17 cell differentiation, focal adhesion, and other pathways (Figure 3B). RNA-seq analysis showed that miR-150_R-1 (R-1 means that the miRNA-seq (detected) is one base shorter than known rep-miRNAseq on the right side) was the most significant miRNA among all DE miRNAs (*p* = 1.16 × 10^−6^) (Supplementary Table S4). According to the prediction of target genes using TargetScan and miranda software, there were 19 DE target genes of miR-150_R-1 (Figure 3C).

### Analysis of DE genes in the blood of NC and RP groups using qRT-PCR and RNA-seq

**Figure 4:**
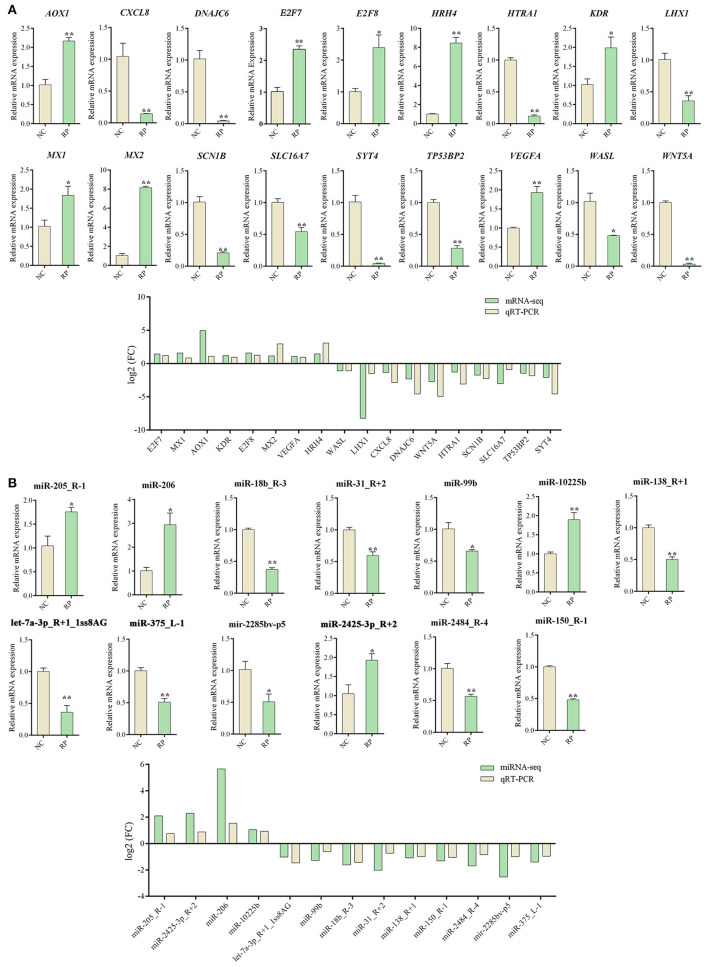
qRT-PCR Validation of DE genes in NC and RP. **(A)** qRT-PCR analysis of DE mRNA expression and comparison of |log2 FC| expression levels of mRNA-seq and qPCR. **(B)** qRT-PCR analysis of DE miRNAs expression and comparison of |log2 FC| expression levels of miRNA-seq and qRT-PCR. Data represent the mean ± SEM. *p < 0.05, **p < 0.01. FC, fold-change; qRT-PCR, quantitative reverse transcription PCR; NC, fetal membranes of normal discharge; RP, retained placenta.

qRT-PCR validation of 18 DE mRNAs and 13 DE miRNAs was performed to verify the accuracy of the RNA-seq results (Figure 4). All selected mRNA and miRNA expression patterns differed significantly between the NC and RP groups (*p* < 0.01, *p* < 0.05). The expression trends of mRNA and miRNA followed those of RNA-seq, indicating that the RNA-seq results were reliable for identifying DE mRNAs and miRNAs.

### Expression levels of EPAS1 and miRNA-150_R-1 in NC and RP groups and transfection efficiency of miRNA-150_R-1 mimic in BEND Cells

**Figure 5:**
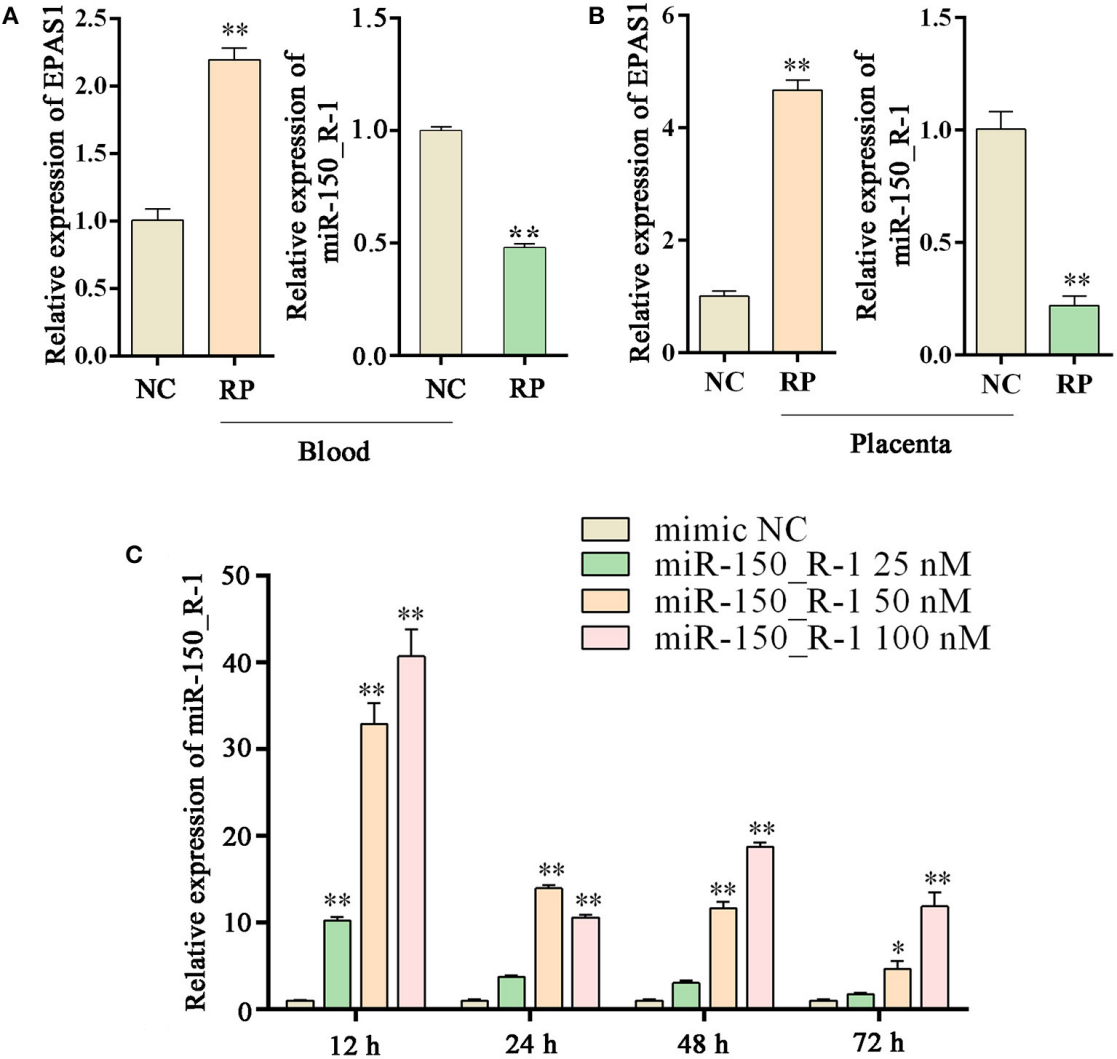
Changes in *EPAS1* and miRNA-150_R-1 in blood and placental tissues and transfection efficiency of miRNA-150_R-1 in BEND cells. **(A)** *EPAS1* and miR-150_R-1 mRNA expression levels in NC and RP blood. **(B)** *EPAS1* and miR-150_R-1 mRNA expression levels in NC and RP placental tissues. **(C)** At 12, 24, 48, or 72 h and final mimic concentrations of 25, 50, or 100 nM, the transfection efficiency of the mimic was verified using qPCR, with mimic NC used as a control. Data represent the mean ± SEM. *p < 0.05, **p < 0.01. NC, fetal membranes of normal discharge; RP, retained placenta.

The expression of *EPAS1* mRNA in the blood of the RP group was significantly upregulated compared to that in the NC group. The expression of miR-150_R-1 mRNA in the blood of the RP group was significantly downregulated compared to that in the NC group. miR-150_R-1 and *EPAS1* showed opposite expression trends (Figure 5A). The expression of *EPAS1* and miR-150_R-1 in the placental tissue was consistent with that in the blood (Figure 5B).

Based on the qRT-PCR analysis, the expression level of miR-150_R-1 mRNA was increased to varying degrees at different periods in BEND cells, but its transfection efficiency was highest at 12 h. At 12 h and a final mimic concentration of 25 nM, miR-150_R-1 mRNA expression was significantly increased by ~10-fold (*p* < 0.01). When the final mimic concentration was 50 nM, the expression of miR-150_R-1 mRNA was significantly increased by ~34-fold (*p* < 0.01). When the final mimic concentration was 100 nM, the expression of miR-150_R-1 mRNA was significantly increased by ~41-fold (*p* < 0.01) (Figure 5C). Because the final mimic concentration increased exponentially but the transfection efficiency did not, 12 h and 50 nM were selected as the optimal conditions.

### HIF-1/ErbB pathway in BEND cells is regulated by miRNA-150_R-1

**Figure 6:**
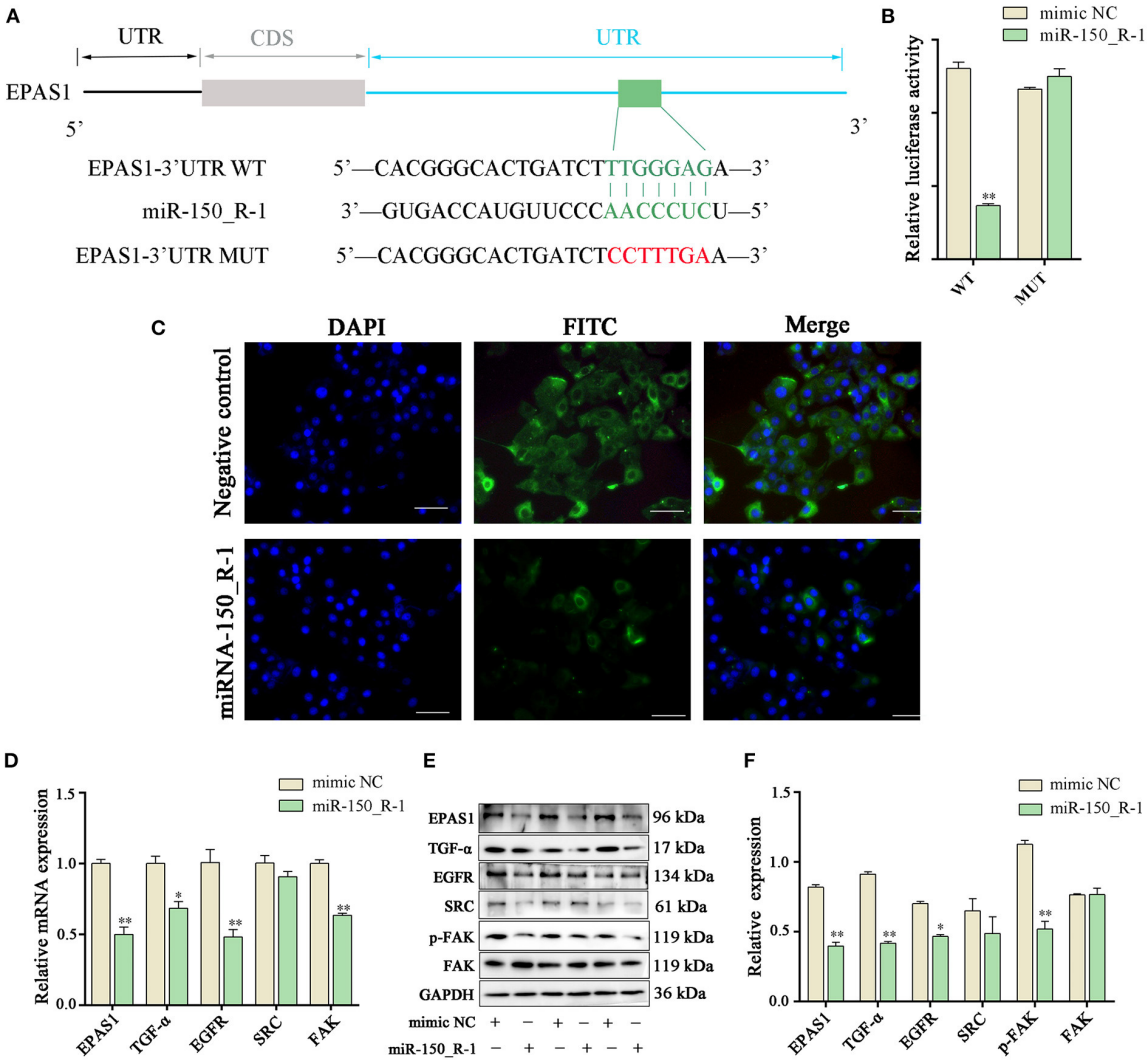
EPAS1 is regulated by miRNA-150_R-1. **(A)** Binding site of EPAS1 and miR-150_R-1. **(B)** Luciferase activity in BEND cells after co-transfection with miR-150_R-1 mimic (50 nM) or mimic NC (50 nM) and pmirGLO-EPAS1 3′-UTR-WT (400 ng) or pmirGLO-EPAS1 3′-UTR-MUT (400 ng). **(C)** Immunofluorescence analysis of EPAS1 in NC mimic and miR-150_R-1 mimic groups. The nuclei were stained with DAPI (blue). Magnification: 100 × . **(D)** Expression levels of mRNAs in HIF-1/ErbB pathways analyzed using PCR. **(E)** Strip chart of proteins in the HIF-1/ErbB pathway by western blotting. **(F)** Grayscale value of the western blotting strip map. Data represent the mean ± SEM. *p < 0.05, **p < 0.01. UTR, untranslated region; CDS, coding sequence; MUT, mutant type; WT, wild-type.

Binding seed sequences in aligned EPAS1 3′-UTR and miR-150_R-1 were predicted using TargetScan and miranda bioinformatics software. The EPAS1-MUT group showed six mutated nucleotides in the 3′-UTR of EPAS1 (Figure 6A). The activity of the luciferase reporter in the EPAS1 3′-UTR-WT+miR-150_R-1 mimic group was significantly lower than that in the EPAS1 3′-UTR-WT+mimic NC group after transfection with the miR-150_R-1 mimic (*p* < 0.01) (Figure 6B). In contrast, there was no significant difference between the mimic NC and miR-150_R-1 mimic groups in the EPAS1 3′-UTR-MUT group (*p* > 0.05). These data confirm that EPAS1 was specifically targeted for inhibition by miR-150_R-1 in the BEND cells.

Immunofluorescence analysis showed that when BEND cells were transfected with miR-150_R-1 mimic or mimic NC, the nuclei stained blue, and EPAS1 was localized in the cytoplasm of BEND cells, showing green fluorescence. Furthermore, upregulation of miR-150_R-1 expression reduced the fluorescence intensity of EPAS1. These results indicate that the miR-150_R-1 mimic was successfully transfected into BEND cells, and the fluorescence intensity of EPAS1 was attenuated by targeted binding of miR-150_R-1 (Figure 6C).

The expression of related molecules in the HIF-1/ErbB signaling pathway was detected using qRT-PCR and western blotting to explore the regulation mechanism of RP by the miR-150_R-1/EPAS1 axis. qRT-PCR showed that the mRNA expression levels of *EPAS1, TGF-*α, *EGFR*, and *FAK* were significantly decreased in the miR-150_R-1 mimic group compared with those in the mimic NC group (*p* < 0.05, *p* < 0.01) (Figure 6D). The western blotting results indicated that the protein expression levels of EPAS1, TGF-α, EGFR, and p-FAK were significantly decreased in the miR-150_R-1 mimic group compared with those in the mimic NC group (*p* < 0.05, *p* < 0.01) (Figures 6E,F). However, the FAK protein expression level did not significantly change (*p* > 0.05). Although SRC expression decreased, the difference was not significant (*p* > 0.05).

## Discussion

The fully developed cow placenta is composed of the maternal and fetal placentas. During pregnancy, the surface of the maternal placenta forms a raised uterine caruncle, and the surface of the fetal placenta forms a raised fetal cotyledon (15). The fetal cotyledon villi tightly wrap the maternal uterine caruncle to form a dense and complete placental structure; a tight connection between these structures is likely to cause RP in dairy cows (16). In this study, clinical symptoms of RP included the failure of the fetal placental cotyledon villi to shed spontaneously from the caruncle crypts of the maternal uterus after delivery of the fetus, and parts of the fetal placenta and uterine caruncle were tightly connected by adhesion and tight junctions. The fetal membranes could not be discharged within the physiological time limit of the third stage of labor. Therefore, cell-cell junctions are critical for RP. We found that the normally discharged placental villi were intact and rich in BVs, whereas the PR placental villi were scattered and shed from the BMs. Additionally, the trophoblast surface of the normally discharged placenta had many neatly arranged microvilli, and the BMs of the villi were intact. In contrast, the placenta in RP showed short and sparse villi, and the BMs of the villi were thickened. Under normal conditions, villous trophoblasts are mainly composed of cytotrophoblasts, syncytiotrophoblasts, and intermediate trophoblasts (16). In the presence of hypoxia, cytotrophoblasts continuously form syncytiotrophoblasts to repair the damaged syncytium layer, causing a thickening of the BMs of the villi (17, 18).

During pregnancy, the uterus-placental circulation-fetus guarantees normal growth and development of the fetus in the uterus (19). The placenta is important for material exchange between the fetus and mother, and the uterine artery is an important bridge (18). The placental villi provide blood to the fetus, and the umbilical artery is the only life channel for the fetus to obtain nutrients and excrete metabolites from the placenta (20). After delivery, the blood supply to the fetal placenta terminates, the villi of the maternal uterine caruncle embedded in the fossae begin to deteriorate, and the tension of the embedded part gradually decreases (21). The closely connected part of the uterine caruncle and fetal cotyledon quickly separate, and the fetal membranes separate naturally. If maternal placental villi and fetal placental villi do not separate over time because of hyperemia, placental hypoxia occurs, the tension of the embedded part is not reduced, and most fetal cotyledons that are closely attached to the uterine caruncle cannot be separated (22). In this study, we predicted the following causes of placental hypoxia: (1) during pregnancy, poor ventilation in the livestock barn, overcrowding between livestock, and lack of exercise leads to blocked circulation in the blood and causes placental hypoxia; (2) during delivery, there is no designated delivery room, inappropriate midwifery, and no quiet environment, causing stress responses that lead to placental hypoxia (23); and (3) normal contraction of the uterus is conducive to the expulsion of the fetus and fetal membranes, but increased activity of the uterus, particularly tonic contraction, causes capillaries of the villi of the uterus caruncle to be severely congested, leading to placental hypoxia (24). Therefore, placental hypoxia is a key factor in RP.

miRNAs exert their functions by regulating transcriptional proteins and play crucial roles in placental development (6). To identify potential genes associated with RP, we performed RNA-seq analysis of blood samples from NC and RP cows. Compared to the NC group, 44 DE miRNAs and 706 DE mRNAs were screened in the RP group. Analysis of the roles of miRNAs in RP showed that DE target genes were mainly involved in biological processes or pathways, such as extracellular matrix (10), cell adhesion (25), immunity (24), and metabolism (16). These factors may directly or indirectly affect the occurrence of RP.

Further analysis of miRNA and mRNA expression levels in the blood samples from the NC and RP groups revealed that *EPAS1* was differentially expressed. EPAS1, or hypoxia-inducible factor-2α (HIF-2α), is a key factor in hypoxia (26). Changes in enzymes or factors caused by hypoxia act mainly through HIF-2α and regulate the expression level of its own and downstream target genes, leading to rapid and accurate responses in tissues and organs (27). The DE gene *EPAS1* was negatively correlated with the most DE miRNA, miR-150_R-1 (*p* = 1.16 × 10^−6^). The expression level of *EPAS1* mRNA was significantly upregulated in the blood and placental tissue of the RP group compared to that in the NC group (*p* < 0.01). The trend in miR-150_R-1 expression showed opposite results. Thus, a strong targeting relationship was observed between miR-150_R-1 and EPAS1. The HIF-1/ErbB signaling pathway corresponding to the target gene *EPAS1* may be involved in regulating cell adhesion. These results suggest that miR-150_R-1 and EPAS1 are involved in the occurrence of RP. When the oxygen transported by the capillaries in the maternal placental villi is too low, leading to placental hypoxia, cell adhesion is enhanced after a series of reactions, causing adherence of endometrial epithelial cells and trophoblast cells and thus causing RP by preventing their separation (25, 28). Therefore, we investigated whether the adhesion of BEND cells is regulated by miR-150_R-1 through the EPAS1-mediated HIF-1/ErbB signaling pathway.

HIF-2α can induce upregulation of erythropoietin, heme oxygenase, and other genes, leading to the production of many red blood cells and increasing oxygen transport throughout the body (29). HIF-2α can also induce upregulation of the receptor TGF-α, promoting the growth of epithelial cells and the formation of local BVs to improve local tissue oxygen supply (30). The signaling pathway of ErbB1, also known as EGFR, refers to multiple processes by which ErbB members dimerize or heterodimerize by binding to numerous signal transducers to promote autophosphorylation and subsequent downstream signaling cascades; regulatory transcription factors activate the transcription of genes that mediate cell migration and adhesion (31). EGFR converts from an inactive monomer into an active homodimer upon binding to EGF or TGF-α (32). Dimerization of EGFR stimulates the activity of intracellular protein tyrosine kinase, which phosphorylates Tyr residues (33). Protein signaling molecules with SRC homology 2 or phosphotyrosine domains dock to these phosphorylated residues (34). FAK or protein tyrosine kinase 2 is a non-receptor and non-membrane-associated protein tyrosine kinase activated at cell-matrix adhesion sites and clustering of integrins by autophosphorylation (at Tyr397), SRC, and other tyrosine kinases (35). FAK regulates cell migration, adhesion, and survival by transferring signals from the extracellular matrix to the cytoplasm *via* integrins (36). Therefore, under hypoxia conditions, both EGFR and FAK in the HIF-1/ErbB signaling pathway may promote cell adhesion, leading endometrial epithelial cells to adhere to trophoblast cells. This may explain why fetal membranes do not detach easily and cause RP. We used miR-150_R-1 to inhibit this process (Figure 6); the results showed that an miR-150_R-1 mimic inhibited the expression of the target gene EPAS1 and reduced the expression levels of TGF-α, EGFR, FAK, and p-FAK in the HIF-1/ErbB signaling pathway. This result sharply contrasts existing knowledge on the HIF-1/ErbB signaling pathway. Our results suggest that the miR-150_R-1-mediated HIF-1/ErbB signaling pathway inhibits the adhesion of endometrial epithelial cells, reducing RP. Aberrant changes in EGFR and FAK phosphorylation are key to understanding the underlying mechanism of RP.

## Conclusions

We demonstrated the relationship between EPAS1 and RP and confirmed that EPAS1 is upregulated in the blood of cows that experience RP. We propose a model of the miRNA-mRNA network mediated by the HIF-1/ErbB signaling pathway to show its regulatory role in RP. EPAS1 is negatively regulated by miR-150_R-1, which then regulates changes in related molecules in the HIF-1/ErbB signaling pathway, particularly abnormal changes in EGFR and p-FAK that affect adhesion between the fetal placental cotyledons and uterine caruncle after delivery, resulting in RP.

## Data availability statement

The data presented in the study are deposited in the GEO repository, accession number GSE214588.

## Ethics statement

The animal study was reviewed and approved by the Animal Protection Committee of Gansu Agricultural University (Lanzhou, China) approved all animal experiments (Approval No.: GSAU-Eth-LST-2021-003).

## Author contributions

CL, YZ, and XZ conceived and designed the study. ZL and QW collected samples. CL and YW performed experiments and analyzed the data. CL wrote the paper. YZ and XZ contributed to the revision of the manuscript. All authors have read and approved the manuscript.

## Funding

This research work was supported by Gansu Province Guiding Science and Technology Innovation Special Project (GSCXZX-2019), Gansu Key Laboratory of Animal Generational Physiology and Reproductive Regulation (20JR10RA563), and Education Science and Technology Innovation Project of Gansu Province (GSSYLXM-02).

## Conflict of interest

The authors declare that the research was conducted in the absence of any commercial or financial relationships that could be construed as a potential conflict of interest.

## Publisher's note

All claims expressed in this article are solely those of the authors and do not necessarily represent those of their affiliated organizations, or those of the publisher, the editors and the reviewers. Any product that may be evaluated in this article, or claim that may be made by its manufacturer, is not guaranteed or endorsed by the publisher.

## Abbreviations

RP: retained placenta
NC: normal discharge of fetal membranes
miRNAs: micro RNAs
nt: nucleotides
3′-UTR: 3′-untranslated
RNA-seq: RNA-sequencing
KEGG: kyoto encyclopedia of genes and genomes
GO: gene ontology
BEND: bovine endometrial epithelial cell
qPCR: quantitative PCR
qRT-PCR: quantitative reverse transcription-PCR
WT: wild-type
EPAS1: endothelial PAS domain protein 1
Mut: mutant type
TGF-α: transforming growth factor-α
EGFR: epidermal growth factor receptor
FAK: focal adhesion kinase
DE: differentially expressed
HE: hematoxylin-eosin
BMs: basement membranes
BVs: blood vessels
FC: fold change
CDS: coding sequence.
