# Supplementary material for: miRNA-150_R-1 mediates the HIF-1/ErbB signaling pathway to regulate the adhesion of endometrial epithelial cells in cows experiencing retained placenta
Source: Front Vet Sci. 2022 Oct 17;9:1037880. doi: 10.3389/fvets.2022.1037880 (PMC9619212; doi:10.3389/fvets.2022.1037880)
Supplement: Supplementary file 1 [file Table_1.DOCX]

**Table S1.** Primer information of mRNAs and miRNAs used for qPCR.

| Gene name | Primer sequence-Forward (5′-3′) | Primer sequence-Reverse (5′-3′) | Amplicon (bp) |
| --- | --- | --- | --- |
| *WASL* | AAGTGCAGTGGTGTTGCTTG | GTCGTCGTCCCAACAAGTCT | 247 |
| *LHX1* | CTTCTTCCGGTGTTTCGGTA | TGTTCTCTTTGGCGACACTG | 224 |
| *CXCL8* | TGCTCTCTGCAGCTCTGTGT | CTTTTCCTTGGGGTTTAGGC | 209 |
| *E2F7* | GCCAGCTCTCAGTGGTTTTC | GACCTTGACATCACCGGACT | 243 |
| *WNT5A* | CCTTCGCCCAGGTTGTAATA | CCGGAACTGATACTGGCACT | 224 |
| *MX1* | GTCCCTGCTAACGTGGACAT | ACCAGGTTTCTCACCACGTC | 155 |
| *AOX1* | AACCTTCCATCCAACACAGC | CACTGGGTCAGGTTCTTGGT | 191 |
| *HTRA1* | ACGCCAAAACCTACACCAAC | GGAAGCTTGCGAAACAGTTC | 214 |
| *KDR* | CCCTTCTTTGAAGCATCAGC | CGTGCTGTTCTTCTTGGTCA | 175 |
| *SCN1B* | GGAAGAGGATGAACGCTTTG | GCCATGTCTCTGTTGGCTTT | 225 |
| *SLC16A7* | CACAAGCCTGGTGGTGTATG | GCTCCACAGGCCAAATACAT | 225 |
| *E2F8* | GTGCAAAGAACCGAGAGAGG | GCACTGGGTGAGAGGGATTA | 167 |
| *MX2* | GCTGGTGCTGAAACTGACAA | GTGATGCCAGGAAGGTCAAT | 225 |
| *TP53BP2* | GAAGGAGTCCCAGACAGCAG | CCAGGAGGCAGAGAAACTTG | 206 |
| *VEGFA* | TCACCAAAGCCAGCACATAG | GCGAGTCTGTGTTTTTGCAG | 251 |
| *SYT4* | GCCTGGACAGCAAAAAGAAG | ACCTCTTTTCCCCCTTCTGA | 202 |
| *HRH4* | TCGAATTCCTGATCCCAGTC | TCCAGGTTGGAAAGAGATGG | 178 |
| *EPAS1* | CACCTTACATCCCCATGGAC | AGGAGGAAGGTGCTGTGAGA | 166 |
| *TGF-α* | TGTGTCTGCCACTCTGGGTA | GACCTCGCAGCAGTGTATCA | 168 |
| *EGFR* | TGCACCATCGACGTCTACAT | TCCTCCATGTCCTCCTCATC | 212 |
| *SRC* | AGGGGAGTTTGCTGGACTTT | TCAGCCACTTTGCATACGAG | 184 |
| *FAK* | GAGTCCAGAAGGCAGGTCAC | ATGCCTGACCTGGGTAGATG | 217 |
| *β-actin* | CTCTTCCAGCCTTCCTTCCT | GGGCAGTGATCTCTTTCTGC | 178 |
| let-7a-3p_R+1_1ss8AG | CGCGGCACTATACAGTCTACTG | Universal reverse* | - |
| miR-99b | AATGCTCACCCGTAGAACCGA | Universal reverse* | - |
| miR-18b_R-3 | AGCCAGCGTAAGGTGCATCTA | Universal reverse* | - |
| miR-31_R+2 | AACACGCAGGCAAGATGCTG | Universal reverse* | - |
| miR-206 | ACCGAGGTTGGAATGTAAGGAAG | Universal reverse* | - |
| miR-205_R-1 | AGGCGCATTCCTTCATTCCAC | Universal reverse* | - |
| miR-138_R+1 | AACGATAAGCTGGTGTTGTGAATC | Universal reverse* | - |
| miR-150_R-1 | AACCTCCTCTCCCAACCCTTG | Universal reverse* | - |
| miR-2425-3p_R+2 | AAGGTTGTTTCGTAGACCCTGC | Universal reverse* | - |
| miR-2484_R-4 | CAGCGCTGAGCTATGATGACTTTGATT | Universal reverse* | - |
| miR-10225b | ACGACAATCGAGCCTGACAGA | Universal reverse* | - |
| mir-2285bv-p5 | AACACGCAGGCAAGATGCTG | Universal reverse* | - |
| miR-375_L-1 | AAGGTTGTTTTGTTCGTTCGGC | Universal reverse* | - |
| U6 | CACGCAAATTCGTGAAGCGTTCCA | Universal reverse* | - |

* Universal reverse was provided by the manufacturer (Evo M-MLV RT Kit with gDNA Clean for qPCR, Accurate Biology, Changsha, Hunan, China).
