# Supplementary material for: miRNA-150_R-1 mediates the HIF-1/ErbB signaling pathway to regulate the adhesion of endometrial epithelial cells in cows experiencing retained placenta
Source: Front Vet Sci. 2022 Oct 17;9:1037880. doi: 10.3389/fvets.2022.1037880 (PMC9619212; doi:10.3389/fvets.2022.1037880)
Supplement: Supplementary file 7 [file Image_1.pdf]

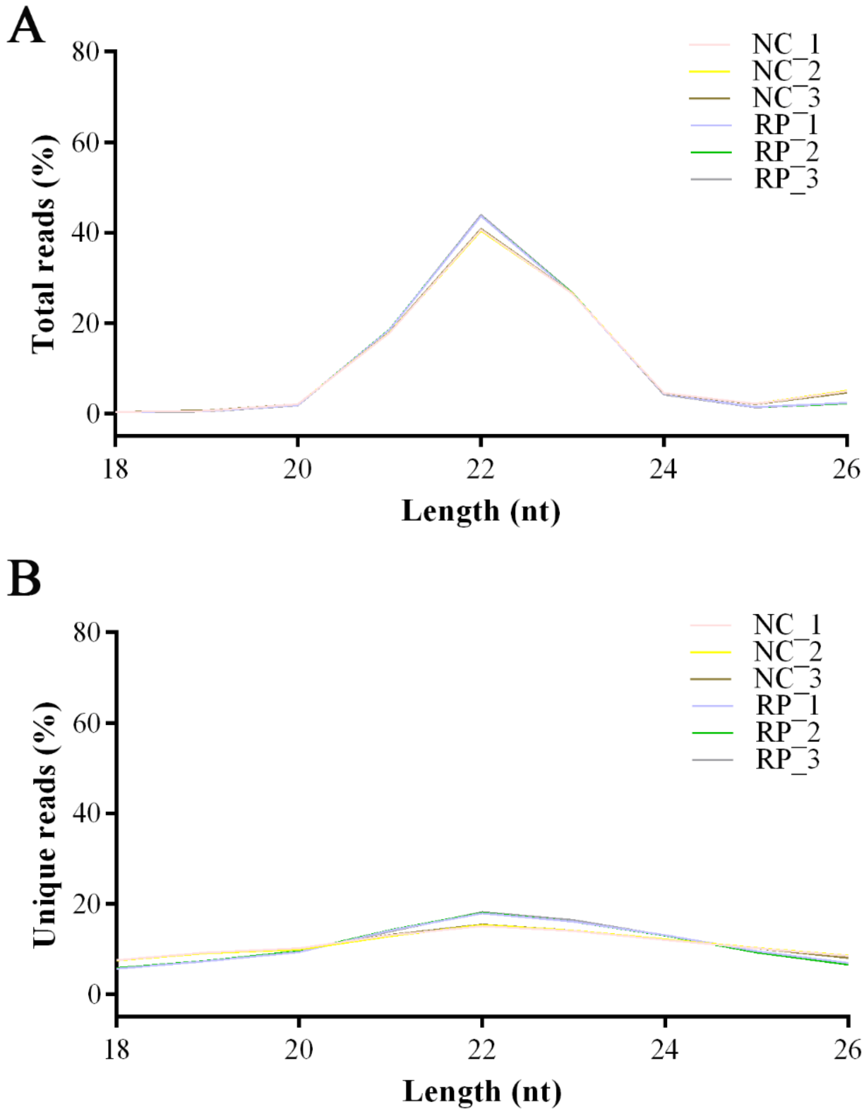

Figure S1. Length distribution of small RNAs (sRNAs). (A) Total sRNAs in different libraries; **(B)** Unique sRNAs in different libraries. NC, fetal membranes of normal discharge; RP, retained placenta.
